# Parent assessment of medical student’s skills in ambulatory pediatrics

**Published:** 2013-09-30

**Authors:** Erika Persson, Christina Haines, Mia Lang

**Affiliations:** Dept. of Pediatrics, Faculty of Medicine and Dentistry, University of Alberta, Edmonton, Alberta

## Abstract

**Background:**

Partnership with parents is a vital part of pediatric medical education, yet few studies have examined parent attitudes towards learners in pediatric settings.

**Methods:**

Questionnaires were used to determine parent and student assessment of professional and clinical skills (primary outcome) and parent attitudes towards 3rd year medical students (secondary outcome) at the University of Alberta. Chi Square, Kendall’s Tau and Kappa coefficients were calculated to compare parent and student responses in 8 areas: communication, respect, knowledge, listening, history taking, physical examination, supervision, and overall satisfaction.

**Results:**

Overall satisfaction with medical student involvement by parents was high: 56.7% of all parents ranked the encounter as ‘excellent’. Areas of lesser satisfaction included physician supervision of students. Compared to the parent assessment, students tended to underrate many of their skills, including communication, history taking and physical exam. There was no relationship between parent demographics and their attitude to rating any of the students’ skills.

**Conclusions:**

Parents were satisfied with medical student involvement in the care of their children. Areas identified for improvement included increased supervision of students in both history taking and physical examination. This is one of the largest studies examining parent attitudes towards pediatric students. The results may enhance undergraduate curriculum development and teaching in pediatric ambulatory clinics and strengthen the ongoing partnership between the community and teaching clinics.

## Introduction

A patient’s decision to have learner involvement in their care balances a need for personal privacy with a willingness to contribute to the education of future physicians.^1^ Published studies have examined patient attitudes towards learners in a variety of settings including obstetrics and gynecology,^1^ dermatology,^2^ family practice,^3^,^4^,^5^ internal medicine,^6^,^7^,^8^ and surgery.^9^ Many of these studies have shown an overall positive attitude towards learners despite increased length of appointments,^1^,^3^ clinically inexperienced students,^1^ unfamiliarity with the role of medical students,^1^ and the fact that many are not forewarned that a medical student will be present for the encounter.^3^ Nevertheless, the quality of some consultation visits is improved with the presence of students.^1^ Patient demographic factors have not been shown to influence patient attitudes towards medical students.^3^ Some patients view themselves as an important tool in medical education, acting as teachers and experts in their own conditions.^3^ Patient satisfaction with clinical encounters also improves compliance and promotes ongoing utilization of health care services.^10^

In many centres training of medical students in pediatrics is completed in ambulatory and in-patient settings. Parental consent to allow student participation in the clinical encounter is an important component required for students to acquire pediatric clinical skills. However, few studies have examined parent attitudes towards learners in either ambulatory or in-patient pediatric settings. In a 1982 study, parents reported that they were satisfied with the care provided by medical students to their children, despite long appointments (*n* = 24).^11^ Another small study (*n* = 11) found that mothers preferred encounters where the medical students demonstrated patient-centred communication skills, including actively seeking parental perceptions and understanding of disease.^12^ One pediatric in-patient study (described in a Letter to Medical Education), reported that parents were highly supportive of medical student involvement in both history taking (93%) and physical examination (86%), but student supervision was a concern.^13^ Parents also thought that communication and self-awareness skills are important to foster in medical students.^14^,^15^ Patients and caregivers can provide invaluable feedback to their health care providers, including students.^15^ Thus, the primary objectives were to examine medical student self-assessment of their skills and compare these to parent assessment of the medical student. Our secondary objective was to determine parent attitudes towards medical students in the pediatric ambulatory setting and to examine if demographic factors of either parents or students could account for these attitudes.

## Methods

### Population and setting

The study population included third year medical students at the University of Alberta (class of 2008) in their first clinical clerkship year and parents of pediatric patients attending the four established general pediatric ambulatory teaching clinics in Edmonton, Alberta, Canada (city population approximately 750,000).

Third year medical students at the University of Alberta complete a 6-week rotation in pediatrics consisting of 3 weeks of in-patient and 3 weeks of ambulatory care (in variable order). Students complete their entire 3 weeks of ambulatory training at the same clinic where the patient population (newborn to age 17 years) includes well-child checks, follow-up of patients with chronic/and or complex medical problems, and acute emergency visits. As a standard practice in the pediatric teaching clinics, parents are informed at registration or when the nurse brings them into the clinic room that they may be seen first by a medical student but that the student would review the material with the physician who would then come into the room and re-review the information with the family. A parent may choose not to have a student present, in which case these families were excluded from the study, but were still seen by the physician for full level of care. Thus, a clinical encounter consists of the medical student taking the history and performing the physical examination, either independently or with the pediatrician in the examining room. If the pediatrician does not directly observe the history or physical exam, the student summarizes the encounter (either inside or outside the examining room); then the pediatrician double-checks the physical exam and key historical information. The pediatrician and medical student close the encounter with a summary of the visit and develop a management plan with the patient and the parents.

### Study design

Over the course of one academic year (September 2006 – August 2007) the entire third year medical class was approached to participate. All students (*n* = 129) were informed of the study by the principal investigators on the first day of their pediatric clerkship rotation. Parents were informed of the study by the clinic staff upon registration of their children to the clinic. To minimize selection bias, all parents whose child was due to be seen by a medical student were approached to participate in the study. Both medical students and parents were provided information sheets describing the purpose of the study. A questionnaire was used to survey student and parent demographics, parent attitudes towards medical student participation in the care of their children, and parent and student assessment of student clinical and communication skills. Clinical preceptors did not have access to the completed student or patient evaluation forms and the results of the questionnaires did not affect student evaluations. Both students and parents were aware of this prior to consenting to participate. To account for potential variability in level of difficulty of patient/parent encounters, we aimed to have five parent/patient encounters per student. Ethical approval for this study was obtained from the Health Ethics Research Board at the University of Alberta prior to the study.

### Development and administration of questionnaires

The authors developed the questionnaires based on the clinical objectives and standardized evaluation forms used by the University of Alberta pediatric clerkship, as well as the CanMEDs (Canadian Medical Education Directions for Specialists) roles for professional and clinical skills in postgraduate medical training. The information sheet, consent form and questionnaires were tailored to a Grade 8 English reading level and were designed to be completed in less than 10 minutes. A 5-point rating scale for survey questions was used from 1 = not at all/very poorly to 5 = completely/excellent. The questionnaire was piloted on 10 parents and 5 medical students prior to the study. The University of Alberta, Division of Studies in Medical Education (DSME) provided a computer-generated 4-digit identification code for each medical student. These codes provided anonymity of student demographics and assessments as well as a method to match parent-student encounters. Only the DSME office and an assistant to the pediatric undergraduate medical education program had access to the codes; the authors and all preceptors were blinded to the codes. Parents and students were informed the code would only be broken if a harmful action was indicated on a questionnaire. Parents likewise completed the questionnaires anonymously.

Parents were asked to evaluate students in 8 clinical domains: communication, respect, knowledge, history taking, physical examination, listening, preceptor supervision (for both history and physical exam) and overall satisfaction. Parents were also asked if their child had previously seen a medical student and if they would see one in the future. If a patient was sufficiently mature, they either completed the forms independently of their parent or with their assistance. Students were asked to evaluate their own performance in the same 8 clinical domains as well as to identify what they thought the corresponding parent assessment was in each of the domains. Demographic data were collected for both students and parents.

### Statistical analysis

To minimize data entry errors, two individuals (EP and a research assistant) independently entered all questionnaire data into separate Microsoft Excel spread sheets. The data were cleaned into a final data set by author CH, with help from another research assistant. The statistical analysis was completed using SPSS (Version 15.0 for Windows) statistical software and the assistance of a biostatistician (CH). Summaries (mean, standard deviation, median and ranges) were obtained for continuous variables (such as age) while frequencies and proportions were obtained for categorical variables (such as gender). Responses to the 5-point rating scale were treated as both continuous and categorical outcomes. Chi Square, Kendall’s Tau and Kappa coefficients were calculated to compare parent and student responses in the 8 clinical domains including overall encounter satisfaction; a *p*-value < 0.05 indicates a significant agreement between the parent and student assessments. Analysis was done separately for each encounter as well as for all encounters combined.

## Results

### Student and parent participation

**Figure 1:**
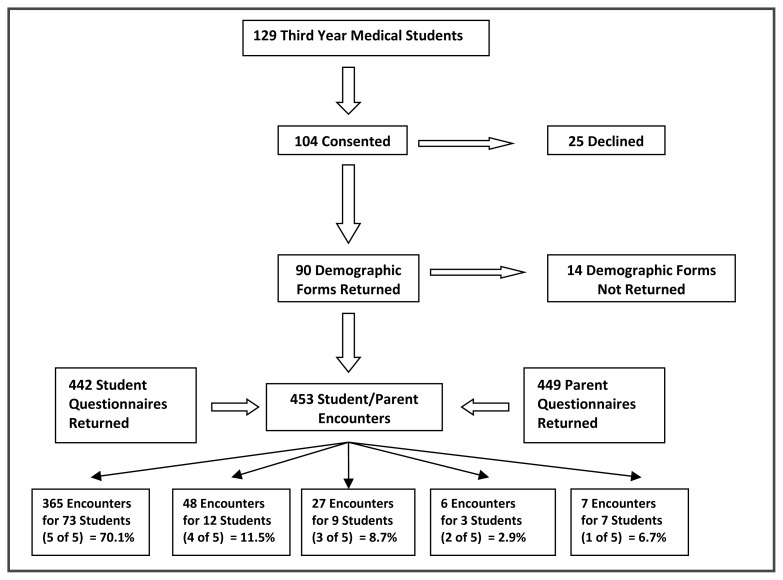
Flowchart summarizing numbers of students and parents involved in the study.

Just over 80% of the third year medical class (104/129) consented to participate, with 90 returning completed demographic forms. The parent participation rate could not be calculated due to logistical difficulties with limited staff and large patient volumes at each ambulatory clinic; nevertheless, 453 parents consented to participate (Figure 1). The number of students and parents participating at two of the four ambulatory clinics was low, however all four ambulatory clinics had similar results in all categories. Therefore, a regional comparison was not completed between the clinics.

### Student and parent encounters and demographics

Of the 453 possible parent-student encounters, 449 parent and 442 student encounter forms were returned. All possible encounters were included when determining frequencies/proportions. The mean number of student-parent encounters was 4.2 (*SD* = 1.2); of the 104 students, 73 (70.1%) achieved the goal of 5 encounters while only 19 (18.3%) had 3 or fewer encounters (Figure 1). Table 1 provides parent and student demographic characteristics of the study population. The mean age of the students was 24.5 years (*SD* = 2.0 years, range 22–34 years).

### Parent attitudes

Parent satisfaction with medical student involvement in the clinical encounter was very high: 385 (84.9%) parents ranked their overall satisfaction as 4 (good) or 5 (excellent); only 5 (1.1%) parents assessed the encounter as 1 (very poor) or 2 (poor). Student overall satisfaction was similar with 337 (74.4%) encounters that scored a 4 or 5 and only 8 (1.8%) encounters were scored 1 or 2 by the students. Parent and student responses to the remaining study questions are represented in Table 2. There was no evidence that parent or student demographic factors (Table 1) accounted for parent attitudes to students in the ambulatory setting, with *p*-values > 0.05 for all corresponding Chi-square tests. When asked if parents would be willing to see a medical student again in the ambulatory setting 420 (93%) said “yes”, 1 (0.2%) said “no”, 25 (6%) were “unsure” and 7 (2%) did not answer the question. The reasons for being unsure or not seeing a medical student again included increased length of appointments, lack of supervision of students, and feeling uncomfortable with the medical student.

### Parent and student clinical skills assessment

Analyses of the results obtained from the individual encounters and all encounters combined were very similar; only the results using all encounters combined are presented. The type of clinical encounter (health maintenance visit, consult, acute illness visit) made no difference to the students’ self-assessment or to the parents’ evaluation. Seven of the eight clinical domains were rated quite high by both parents and students. With respect to student listening, 423 (93%) parents and 356 (79%) students evaluated the domain as 4 (very good) or 5 (excellent). However, when asked about physician supervision of medical students in history taking and physical examination, both parents and students had lesser degrees of satisfaction (Table 2). Of the 372 parents who answered this question, 78 (21%) rated supervision as 1 or 2; 81 of the 453 parents (18%) did not answer this question. Students were asked separate questions about supervision of history taking and physical examination. In regards to supervision of history taking, 13 of the 382 returned responses were 1 or 2 (2%), while 71 of the 453 responses (16%) were left blank. Similar results were found for physical examination supervision: 16 of 374 responses were 1 or 2 (4%), and 79 (17%) of the 453 total responses were left blank. Reasons for non-responses were not collected. The students’ self-assessment scores (mean ± *SD*) for communication (4.0 ± 0.7), respect (4.3 ± 0.7), knowledge (3.5 ± 0.7), history (4.0 ± 0.7), physical (3.8 ± 0.8) and listening (4.1 ± 0.7) were not statistically different from how they thought the parents rated these skills. Student experience (prior education, work experience, students with children, or stage in clerkship) made no difference in parent attitudes, parent assessment or student self-assessment (Pearson’s Chi Square p-values *p* > 0.05). When comparing parent and student responses, two of the four questions pertaining to communication had a *p*-value < 0.05, and thus indicated that there was agreement in scores between the two groups; likewise one of the questions in the knowledge section indicated agreement, as well as agreement for history and physical exam, listening, supervision of physical exam, and overall satisfaction (Table 2).

## Discussion

We conducted one of the largest studies to date examining patient or parent attitudes to pediatric learners. Parents in Edmonton were very satisfied with medical student involvement in the care of their children in the ambulatory setting. The high degree of satisfaction with medical students in pediatrics was not surprising as our results were similar to those found by previous studies,^1^–^8^ although the students tended to score themselves lower compared to the parents, particularly in the areas related to respect and knowledge. This may be because the students were less confident in these areas and thus underestimated their own abilities.

No demographic factors of either parents or students were identified in this study that would help to predict attitudes. While these findings concur with some studies,^6^ others have suggested that acceptance of medical students may be associated with older patient age.^1^ Past studies have not found a relationship between prior encounters with medical students and acceptance of medical students.^3^,^8^ Our results confirmed these findings, with 75.7% of parents having had a previous encounter with a medical student and 93% agreeing to see a medical student again. In addition, parent attitudes were not associated with students’ increasing clinical experience. Parents were equally satisfied with 3^rd^ year medical students in their first, or their last, clinical rotation. This may be the result of patient-centred care and clinical skills training provided in the pre-clerkship years (1^st^ and 2^nd^ year) at the University of Alberta.

In assessment of clinical domains, both parents and students had high ratings of student performance. Parents highly valued communication skills,^13^, ^14^, ^15^ and in two areas of communication (lack of jargon, parent opportunity to speak), parents and students agreed that this skill was performed well. However, students self-ratings of their abilities to ask questions related to the chief complaint and provide information was lower than the parents’ assessments of these skills, resulting in a disagreement between these two groups in these communication areas. Likewise, the medical students rated their respect and most areas in knowledge lower than the parents did. These results suggest that in many areas, medical students may undervalue their contribution to quality pediatric care.

Of concern, almost one fifth of the parents thought that there was poor or very poor supervision by the attending pediatrician (a further 18% of parents did not respond to this question). Most students were satisfied by the amount of supervision for their history taking and physical exam, but a small percentage were not, and like the parents, many students did not answer this question. The relatively high non-response rate to this question may be caused by students and parents feeling uncomfortable about commenting on the physician’s behaviour or, perhaps because the physicians did the history and/or physical exam themselves and thus there was no need to answer the supervision questions. Despite this, the majority of parents in our study indicated they would agree to having a medical student see their child again. Nevertheless, this perceived lack of supervision is concerning as it could influence future participation of parents in medical student learning as well as the learning environment for the students. It also raises the question of supervision expectations (what kind (direct/indirect) and how much) of both the parents and the students. When possible, clinical preceptors should observe the students take a history and physical exam directly, particularly in the beginning of the student’s rotation. As the student progresses in clinical experience, they and the parents should be told that the student will do some, or all, of the child’s history and physical exam independently and that it will be reviewed with the preceptor who will double-check the findings. Although costly, other options, especially for designated teaching clinics, would be the installation of one-way mirrors and/or cameras for video-recording; guidelines for their use must be understood by the clinic and families however, especially in regards to confidentiality with any form of recording equipment. In our teaching clinics, student supervision and teaching does increase the length of the appointment, by as much as 5 – 30 minutes depending on the type of clinical encounter. Despite this increased length of appointments, rarely did parents cite this as a reason not to see a medical student again.

Unfortunately, not all limitations of this study could be overcome. Despite an extensive literature search, a standardized validated survey tool was not found that could best address our primary objective; thus we created our own tool based on the student evaluation forms used for many years at this medical school. Prior to study enrollment, a pilot trial was conducted to ensure questionnaire clarity and ease of administration within the ambulatory environment; however, due to resource, financial and time restraints we were unable to validate the questionnaire. There was a selection bias in that the questionnaire was offered only in English, limiting participation to those who could read English at a grade 8 level, and possibly causing the questions to be misunderstood by those who have English as a second language. The participation rate could have been increased if the survey was offered in additional languages, as Edmonton has a large multicultural population. Due to logistical constraints at the ambulatory sites we were unable to determine the consent and participation rate of parents in this study. A participation bias had the potential to exist within the study as parents who had either very positive or negative attitudes towards students could have chosen to consent. Despite these limitations, this was one of the largest studies examining parent attitudes and overall satisfaction with medical students in ambulatory pediatrics was quite high.

## CONCLUSIONS

Parents in this study were very satisfied with medical student involvement in the care of their children in the pediatric ambulatory setting. As one of the largest studies addressing attitudes towards learners, our results are encouraging as we rely on patient and parental support and consent for medical student involvement for the training of future physicians. Supervision of students is an important aspect of clinical education and our results suggest that this may be an area for improvement. This would not only benefit the students, but could continue to promote a cooperative relationship between ambulatory pediatric teaching clinics and the community.

## Figures and Tables

**Table 1:** Parent and student demographics.

Parent Demographics	n*	%	Student Demographics	*n*†	%

**Age (years)**			**Gender**		
14–19	10	2.2	Male	39	43.3
20–30	124	27.6	Female	49	54.4
31–40	223	49.7	Not Indicated	2	2.2
41–50	68	15.1	**Ethnicity**		
>51	9	2.0	Caucasian	47	52.2
Not Indicated	15	3.3	Asian	23	25.6
**Gender**			Middle Eastern	4	4.4
Male	60	13.4	First Nations	1	1.1
Female	378	84.2	Other	8	8.9
Not Indicated	11	2.4	Not Indicated	5	5.6
**Child Age**			**First Language**		
Newborn (1–30 days)	15	3.3	English	79	87.8
Infant (1–12 months)	113	25.2	Other	10	11.1
Toddler (13–35 months)	91	20.3	Not Indicated	1	1.1
Pre-school (36–56 months)	50	11.1	**Prior Degrees**		
School Age (5 to 12 years)	116	25.8	BSc	53	58.9
Adolescent (13–17 years)	31	6.9	BA	2	2.2
Multiple Children at Visit	31	6.9	Engineering	3	3.3
Not Indicated	2	0.4	Other	8	8.9
**Education Level**			Not Indicated	24	26.7
<Grade 10	20	4.5	**Prior Employment**		
Grade 11–12	80	17.8	Yes	47	52.2
University/College	341	75.9	No	38	42.2
Not Indicated	8	1.8	Not Indicated	5	5.6
**Family Income**			**Marital Status**		
< $20,000	32	7.1	Single	73	81.1
$20–49,000	67	14.9	Married	14	15.6
$50–74,000	105	23.4	Common Law	3	3.3
>$75,000	224	49.9	**Students as Parents**		
Not Indicated	21	4.7	Yes	3	3.3
			No	81	90.0
			Not Indicated	6	6.7
**Health Care Provider - Self**					
Yes	67	14.9			
No	375	83.5			
Not Indicated	7	1.6			
**Health Care Provider - Family**					
Yes	116	25.8			
No	317	70.6			
Not Indicated	16	3.5			
**Ethnicity**					
Caucasian	279	62.1			
Asian	25	5.6			
Middle Eastern	5	1.1			
African	5	1.1			
First Nations	25	5.6			
Other	17	3.8			
Not Indicated	93	20.7			
**First Language**					
English	383	85.3			
French	6	1.3			
Other	54	12.0			
Not Indicated	6	1.3			
**Prior Encounter with Medical Student**					
Yes	340	75.7			
No	80	17.8			
Unknown	29	6.4			

* Of the 453 consenting parents, 449 parent demographic forms were returned.

† Of the 104 consenting students, 90 student demographic forms were returned

**Table 2:** Comparison of parent and student evaluations

Domain	Parent Evaluations (*n* = 449 returned)		Student Evaluations (*n* = 442 returned)		Kendall’s Tau	p-Value
	Parent Question	Mean ± *SD* (scale 1–5)	Student Question	Mean ± *SD* (scale 1–5)		
Communication	Did the student use words that were easy to understand ?	4.8 ± 0.8	How do you think parents rated your level of communication?	3.9 ± 0.7	0.06	0.01*
	Did the student give you a chance to talk ?	4.8 ± 0.5			0.08	< 0.01*
	Did the student ask questions related to your concerns ?	4.7 ± 0.6			0.05	0.05
	How well did the medical student provide information?	4.3 ± 0.8			0.05	0.23
Respect	Did the medical student seem caring ?	4.7 ± 0.6	How do you think parents rated your level of respect?	4.1 ± 0.7	0.06	0.20
	Did the medical student treat you with respect?	4.8 ± 0.4			0.03	0.14
	Did the medical student treat your child with respect?	4.9 ± 0.4			0.04	0.06
Knowledge	Did the student ask questions related to your concerns ?	4.7 ± 0.6	How do you think parents rated your level of knowledge?	3.5 ± 0.7	0.02	0.38
	How well did the medical student provide information?	4.3 ± 0.8			0.02	0.57
	How knowledgeable did the medical student seem in answering your questions?	4.3 ± 0.8			0.11	< 0.01*
History	Did the medical student seem comfortable taking a history?	4.6 ± 0.7	How do you think parents rated your level of comfort with history taking?	3.9 ± 0.6	0.10	0.02*
Physical	Did the medical student seem comfortable doing a physical exam?	4.5 ± 0.7	How do you think parents rated your level of comfort with physical examination?	3.7 ± 0.7	0.19	< 0.01*
Listening	Did the student give you a chance to talk ?	4.8 ± 0.5	How do you think parents rated your level of listening skills?	4.0 ± 0.6	0.06	0.02*
	How well did the medical student listen to you?	4.7 ± 0.6			0.16	< 0.01*
History Supervision	How much direct supervision by the doctor did the medical student have?	3.6 ± 1.4	Do you think you were adequately supervised with your history skills?	4.0 ± 0.8	0.09	0.06
Physical Supervision	How much direct supervision by the doctor did the medical student have?	3.6 ± 1.4	Do you think you were adequately supervised with your physical exam skills?	4.0 ± 0.8	0.13	0.01*
Overall Satisfaction	Overall, how did you feel today about your experience with the medical student?	4.4 ± 0.8	Based on this patient encounter, how would you rate your level of satisfaction with the overall encounter?	3.9 ± 0.7	0.13	< 0.01*
